# 45例晚期非小细胞肺癌EGFR-TKIs治疗获益后出现孤立进展后联合局部治疗的回顾性分析

**DOI:** 10.3779/j.issn.1009-3419.2013.10.03

**Published:** 2013-10-20

**Authors:** 昕 张, 彬 王, 琳 林, 学志 郝, 闪闪 陈, 峻岭 李, 湘茹 张, 远凯 石

**Affiliations:** 100021 北京，中国医学科学院北京协和医学院肿瘤医院内科，抗肿瘤分子靶向药物临床研究北京市重点实验室 Department of Medical Oncology, Cancer Institute/Hospital, Chinese Academy of Medical Sciences & Peking Union Medical College; Beijing Key Laboratory of Clinical Study on Anticancer Molecular Targeted Drugs, Beijing 100021, China

**Keywords:** 肺肿瘤, 表皮生长因子受体酪氨酸激酶抑制剂, 孤立进展, Lung neoplsms, Epidermal growth factor receptor tyrosine kinase inhibitors, Solitary progression

## Abstract

**背景与目的:**

表皮生长因子受体（epidermal growth factor receptor, EGFR）酪氨酸激酶抑制剂（tyrosine kinase inhibitors, TKIs）小分子物质是EGFR敏感突变肺癌患者的主要治疗手段之一。TKIs耐药后有多种表现形式，其中包括孤立性病灶局部进展。本研究对孤立病灶进展患者后继治疗进行分析。

**方法:**

回顾性研究中国医学科学院肿瘤内科45例晚期非小细胞肺癌患者接受EGFR-TKI治疗后出现孤立进展的局部治疗联合TKIs治疗。

**结果:**

45例患者中11例获得部分缓解（partial response, PR）（24%），23例患者疗效为病情稳定（stable disease, SD）（51%），11例病情进展（progressive disease, PD），局部进展后再次TKI联合局部治疗的无进展生存期（progression free survival, PFS）为5.9个月。

**结论:**

在接受EGFR-TKIs治疗的非小细胞细胞肺癌患者出现孤立病灶进展后，局部治疗联合TKIs药物治疗，有可延长患者的无病生存时间。

## Abstract (en)

**Background and objective:**

Epidermal growth factor receptor tyrosine kinase inhibitors (EGFR-TKIs) are routine treatments for non-small cell lung cancer (NSCLC) with *EGFR* mutation. Different clinical modes can be used to treat patients with TKI resistance, one of which is solitary progression. The aim of this study is to analyze available treatments for patients undergoing solitary progression.

**Methods:**

Clinical data of 45 patients with advanced NSCLC who received local radiotherapy combined with EGFR-TKI after solitary progression were reviewed and analyzed.

**Results:**

Among the 45 patients, 11 showed partial response, 23 achieved stable disease state, and 11 experienced progressive disease. The median of progression-free survival (PFS) was 5.9 months.

**Conclusion:**

Local therapies combined with EGFR-TKIs can prolong the PFS of patients with NSCLC who exhibited solitary progression during EGFR-TKI treatment.

表皮生长因子受体（epidermal growth factor receptor, EGFR）酪氨酸激酶抑制剂（tyrosine kinase inhibitors, TKIs）是*EGFR*敏感突变肺癌患者的主要治疗手段之一。这类患者应用TKIs可以获得约70%的客观缓解率及约10个月的无进展生存时间^[1, 2]^。然而在接受一段时间的TKI治疗后，几乎所有患者会出现耐药。TKIs耐药后主要表现为三种模式，一种是爆发进展，表现为疾病控制时间短，肿瘤负荷快速增加，以及患者出现明显的临床症状；第二种是肿瘤缓慢进展，表现为疾病控制时间长，通常超过6个月，肿瘤负荷轻微增加，患者症状较为轻微；第三种是局部进展，表现为孤立性病灶的进展。对于不同部位孤立进展的患者，NCCN指南中推荐了如手术、放疗等局部治疗的方法，同时建议继续使用EGFR-TKIs。一些研究证实晚期非小细胞肺癌（non-small cell lung cancer, NSCLC）患者在EGFR-TKIs治疗受益后出现孤立进展，在继续全身TKIs治疗的前提下进行局部的治疗，可延长患者的中位无进展生存时间及总的生存时间。

## 1 材料与方法

### 1.1 临床资料

本研究回顾性分析了自2007年8月-2012年12月在医科院肿瘤医院内科接受EGFR-TKIs治疗后出现孤立进展接受局部治疗的45例晚期NSCLC患者，其中男性患者16例，女性患者29例，中位年龄56.9岁（39岁-79岁），均为病理组织学或细胞学检查确诊为肺腺癌。

### 1.2 治疗方法

在患者初次接受EGFR-TKIs治疗时，3例为一线治疗，28例为二线治疗，14例为三线治疗。既往接受的化疗药包括：紫杉醇、多烯紫杉醇、吉西他滨、培美曲塞、长春瑞滨、顺铂、卡铂、奈达铂等药物。初次TKIs治疗时，33例患者接受吉非替尼治疗，11例患者接受厄洛替尼治疗，1例接受埃克替尼治疗。全部病例接受在EGFR-TKIs治疗后第一次复查时（治疗后4周）都获得了部分缓解（partial response, PR）或者病情稳定（stable disease, SD）的疗效。TKIs药物均采用常规剂量，即吉非替尼250 mg，每日1次；厄洛替尼150 mg，每日1次；埃克替尼125 mg，每日3次。本组患者出现孤立灶进展时采用的局部治疗为放疗。

### 1.3 评价指标

主要观察患者接受后继治疗后取得的疗效与无进展生存期（progression free survival, PFS）。将患者首次接受EGFR-TKIs治疗至出现孤立灶进展的PFS设为PFS1，将出现孤立进展后继续EGFR-TKIs治疗同时联合接受局部治疗后取得的PFS设为PFS2。疗效评价采用RECIST实体肿瘤评价标准。无进展生存定义为治疗开始至患者出现病情进展或随访结束的时间。

### 1.4 随诊和统计学处理

随诊截止至2013年4月31日。中位随访时间为2.13年（0.19年-4.11年）。统计学分析采用SPSS 17.0软件，使用*Kaplan-Merier*法计算无进展生存。以*P* < 0.05为差异有统计学意义。

## 2 结果

45例患者用药后到出现独立病灶进展的中位PFS1为11（4-59）个月。其中，23例（51%）患者出现中枢神经系统（central nervous system, CNS）转移，22例（49%）患者出现其他系统转移，分别为淋巴结转移8例；骨转移6例；肾上腺转移5例；肝脏转移3例。在出现独立病灶进展后，29例患者接受局部放疗同时继续原EGFR-TKIs药物治疗；16例患者接受局部放疗并换用其他EGFR-TKIs药物治疗。患者基本资料见表 1。

**1:** 患者基线特征 Basic characteristics of all patients

Characteristics	*n*	Proportion (%)
Total	45	
Gender		
Male	16	35.6
Famle	29	64.4
Age		
Median	56.9	
Range	39-79	
Histological type		
Adenocarcinoma	45	100
*EGFR* mutation		
Exon-19	2	4.4
L858R	3	6.7
Unknown	40	88.9
Solitary progression		
CNS	23	51
Lymph node	8	
Bone	6	
Adrenal gland	5	
Liver	3	
EGFR: epidermal growth factor receptor; CNS: central nervous system.		

45例患者在接受后继治疗后进行疗效评价，11例（24%）患者获得PR，23例（51%）患者疗效为SD，11例（24%）进展，PFS2为5.9个月。其中CNS转移的23例患者疗效PR者8例（35%），SD者12例（52%），3例（13%）进展，PFS2为7.6个月。非CNS转移的22例患者，疗效PR者3例（14%），疗效SD者11例（50%），8例（36%）疾病进展（progressive disease, PD），PFS2为4.1个月。发生中枢神经系统进展的患者比非中枢神经系统进展者更能从联合治疗中获益（*P*=0.017，图 1）。后继治疗采用放疗联合继续EGFR-TKIs药物的29例患者，16例（55%）患者疗效为SD，6例（21%）疗效为PR，7例（24%）为PD，PFS2为5.4个月。采用放疗联合其他EGFR-TKIs药物治疗的16例患者，8例（50%）疗效为SD，5例（31%）疗效为PR，3例（19%）为PD，PFS2为6.1个月。疗效分析见表 2。

**1:**
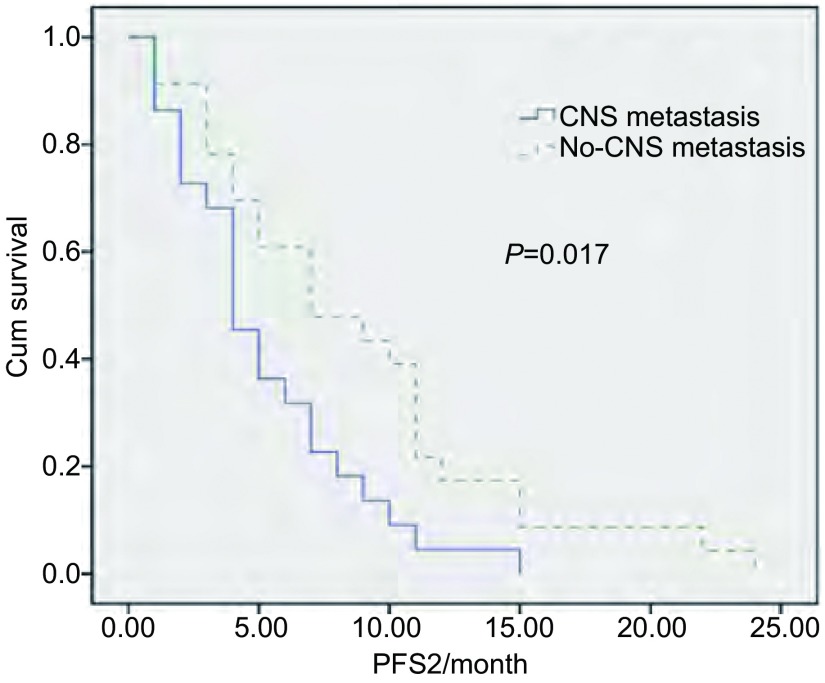
中枢神经系统转移与非中枢神经系统转移患者的PFS2 PFS2 of CNS metastasis and No-CNS metastasis

**2:** 疗效评价 Response of all patients

Patients	Response			PFS2 (month)
	PR	SD	PD	
Total	11 (24%)	23 (51%)	11 (24%)	5.9
CNS metastasis (23)	8 (35%)	12 (52%)	3 (13%)	7.6
No-CNS metastasis (22)	3 (14%)	11 (50%)	8 (36%)	4.1
Continuation-EGFR-TKI+Local therapy (29)	6 (21%)	16 (55%)	7 (24%)	5.4
Switch- EGFR-TKI+Local therapy (16)	5 (31%)	8 (50%)	3 (19%)	6.1
TKIs: tyrosine kinase inhibitors; PR: partial response; SD: stable disease; PD: progressive disease. CNS metastasis *vs* No-CNS metastasis, *P*=0.017. PFS: progression free survival.				

## 3 讨论

本回顾性研究显示，局部放疗联合TKIs，可以延长出现孤立进展灶的晚期NSCLC患者PFS。进展病灶位于CNS的患者，疾病控制率达到87.0%，PFS2为7.6（1-23）个月。非CNS出现进展病灶的患者，疾病控制率为63.6%，PFS为4.1（1-12）个月。后继治疗采用放疗联合继续TKIs药物的患者，疾病控制率为75.9%，中位进展时间为5.4（1.5-10）个月。采用放疗联合其他EGFR-TKIs药物治疗的患者，疾病控制率为81.3%，中位进展时间6.1（1-12）个月。可以看到，在CNS出现孤立进展病灶的患者在疾病控制率和中位进展时间双方面都取得了很好的效果。而后继治疗使用原EGFR-TKIs药物或换用其他EGFR-TKIs药物的疗效相似，无明显差异。

在EGFR-TKIs治疗NSCLC患者出现原发病灶稳定或进一步缩小，同时新出现独立病灶进展的情况下，后续如何选择治疗尚无明确定论。基于其不同的耐药机制，目前认为在EGFR-TKIs治疗进展后继续该药物治疗患者仍可能获益。

T790M突变是目前较被认可的主要耐药机制之一，研究显示约50%的患者经过EGFR-TKIs治疗后出现了二次突变，即T790M突变，也就是EGFR外显子20激酶区存在蛋氨酸代替苏氨酸，导致EGFR结构发生改变，使TKIs与其结合受阻，从而使原来被抑制的磷酸化信号传导过程被重新启动，最终使这类药物无法阻断EGFR磷酸化所介导的信号传导而导致耐药^[3]^。目前存在争议的是T790M突变是先天存在，还是EGFR-TKIs治疗后出现。大多数研究是在EGFR-TKIs出现耐药的患者中发现T790M突变，认为T790M突变是EGFR-TKIs治疗导致；但后来在未经任何治疗的患者标本中也发现T790M突变，所以也有学者推测T790M突变原本就存在，由于这些细胞克隆对EGFR-TKIs抵抗而在治疗后被选择出来。

*Met*基因属于进化上比较保守的生长因子酪氨酸激酶跨膜受体（protein tyrosine kinase, PTK）成员，2007年Engelman等^[4]^首次在细胞系中发现c-MET扩增引起ERBB3信号传递活化是吉非替尼抵抗的重要原因。*c-MET*基因扩增的肿瘤细胞可以绕过被抑制的EGFR磷酸化激酶通路，通过细胞膜上面的c-MET受体磷酸化激酶启动下游信号传导通路（PI3K/Akt通路），从而逃避EGFR-TKIs药物的作用。目前研究^[5]^认为约20%的继发耐药是*c-MET*基因扩增所致。临床前研究发现在对吉非替尼耐药的肺癌细胞系中有*c-MET*基因扩增，并且可以通过阻断MET信号通路而恢复对吉非替尼的敏感性。

为了克服上述机制导致的耐药，仍需要继续抑制EGFR，Riely等^[6]^的研究显示对EGFR抑制剂敏感的肿瘤出现进展后停止EGFR-TKIs治疗可导致肿瘤进展加速。

在肿瘤的发展进展期，癌细胞内部的基因转化可以使部分克隆获得增殖和转移的优势，使之成为同一个体不同病灶对化疗或者TKIs靶向治疗反应不一的异质性来源^[7]^。在本文所回顾的独立病灶进展患者中，即有部分为这种异质性表现。而独立的进展病灶往往可通过放疗等方法得到控制，这可以解释患者在接受放疗后继续使用原有药物取得了较好的疗效。Andrew等^[8]^在近期发表的一项小规模单中心回顾性研究中提出，对进展病灶的局部处理也许可以阻断恶化位点抗性肿瘤克隆的扩散，从而让靶向疗法继续发挥作用。并建议在仅有少量位点发生疾病进展时，可以考虑采用在疾病进展位点采用局部治疗，同时继续TKIs治疗。

在医科院肿瘤医院另一项研究中，初次使用易瑞沙治疗临床获益的患者，经过一段时间后二次接受易瑞沙治疗仍有67%的患者可获益^[9]^，既往的一些其他研究^[6, 10, 11]^中也得到过类似的结果。本研究中的发现独立病灶进展后接受局部放疗并继续原方案治疗，与局部放疗并换用其他EGFR-TKIs药物治疗，在疾病控制率与PFS方面均无明显差异，这一点可能仍旧与同一个体不同病灶对靶向治疗反应的异质性有关。

有研究表明，在吉非替尼失败后应用厄洛替尼可能使患者获益，对此的解释可能为厄洛替尼的血药浓度高于吉非替尼^[12, 13]^，且临床前研究显示厄洛替尼对部分EGFR野生状态的NSCLC细胞亦有一定的抑制作用^[14, 15]^。本研究中有9例接受吉非替尼治疗出现独立病灶进展后停药换用厄洛替尼及局部放疗，取得了77.8%的疾病控制率。因病例数较少，无法进行进一步分析。

## 4 结语

TKIs耐药有多种不同模式，需根据不同耐药模式采用相应的治疗手段。而在临床应用中，TKIs耐药患者后继治疗的个体化选择显得尤为重要。本研究回顾的病例样本较少，所得数据会有一定偏差，尚无法做出一个肯定的结论。大部分患者受条件限制未进行*EGFR*突变情况检测。然而本研究结果仍可提示，在接受EGFR-TKIs治疗的NSCLC患者出现孤立病灶进展的时候，局部放疗联合TKIs药物治疗，可延长EGFR-TKIs药物的PFS。尤其是对于中枢神经系统孤立转移的患者，可取得较好的疾病控制率和PFS。同时，我们将在未来的工作中对此类患者进行进一步的研究随访，也希望有更多的同行提出自己的宝贵经验共同讨论。
